# Cellular senescence and the tumour microenvironment

**DOI:** 10.1002/1878-0261.13268

**Published:** 2022-06-26

**Authors:** Masaki Takasugi, Yuya Yoshida, Naoko Ohtani

**Affiliations:** ^1^ Department of Pathophysiology, Graduate School of Medicine Osaka Metropolitan University (formerly, Osaka City University) Osaka Japan

**Keywords:** anti‐tumour immunity, cellular senescence, senescence‐associated secretory phenotype, senolysis, senomorphics, tumour microenvironment

## Abstract

The senescence‐associated secretory phenotype (SASP), where senescent cells produce a variety of secreted proteins including inflammatory cytokines, chemokines, matrix remodelling factors, growth factors and so on, plays pivotal but varying roles in the tumour microenvironment. The effects of SASP on the surrounding microenvironment depend on the cell type and process of cellular senescence induction, which is often associated with innate immunity. Via SASP‐mediated paracrine effects, senescent cells can remodel the surrounding tissues by modulating the character of adjacent cells, such as stromal, immune cells, as well as cancer cells. The SASP is associated with both tumour‐suppressive and tumour‐promoting effects, as observed in senescence surveillance effects (tumour‐suppressive) and suppression of anti‐tumour immunity in most senescent cancer‐associated fibroblasts and senescent T cells (tumour‐promoting). In this review, we discuss the features and roles of senescent cells in tumour microenvironment with emphasis on their context‐dependency that determines whether they promote or suppress cancer development. Potential usage of recently developed drugs that suppress the SASP (senomorphics) or selectively kill senescence cells (senolytics) in cancer therapy are also discussed.

## Graphical abstract

The senescence‐associated secretory phenotype (SASP), where senescent cells produce a variety of secreted proteins, plays pivotal roles in the tumour microenvironment. SASP remodels the surrounding tissues by modulating the character of adjacent cells and can promote or suppress tumorigenesis. In this review, we discuss the features and roles of senescent cells in tumour microenvironment with focus on their context‐dependency.

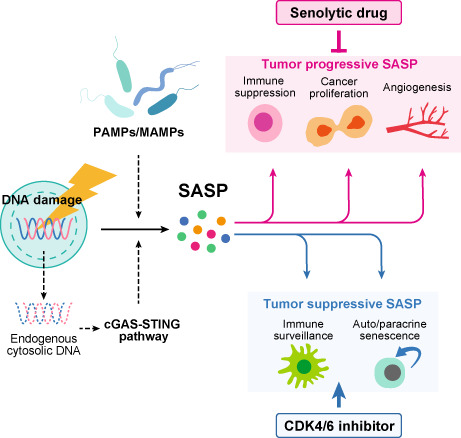

**Abbreviations**
AA: arachidonic acid
BETd: BET family protein degrader
C/EBPβ: CCAAT/enhancer‐binding protein beta
CAF: cancer‐associated fibroblast
CCL2: Cf‐C motif chemokine ligand 2
CCR2: C‐C motif chemokine receptor 2
CDKI: cyclin‐dependent kinase inhibitor
cGAS: cyclic GMP‐AMP synthase
CKIα: casein kinase 1 α
CXCL1: C‐X‐C motif chemokine ligand 1
DAMP: danger‐associated molecular pattern
DCA: deoxycholic acid
EMT: epithelial–mesenchymal transition
GATA4: GATA‐binding protein 4
H3K27: histone 3 Lys27
HFD: high‐fat diet
HSCs: hepatic stellate cells
IFN‐I: type‐I interferon
IL6: interleukin‐6
JAK: Janus kinase
LTA: lipoteichoic acid
MAMP: microbe‐associated molecular pattern
MDSCs: myeloid‐derived suppressor cells
Mϕ: macrophage
NHEJ: nonhomologous end‐joining
NK: natural killer cells
OIS: oncogene‐induced senescence
PAMP: pathogen‐associated molecular pattern
PGE_2_
: prostaglandin E_2_
SASP: senescence‐associated secretory phenotype
SC: senescent cells
STAT: signal transducer and activator of transcription
STING: stimulator of interferon genes
T: T cells
TGF‐β: transforming growth factor‐β
TIS: therapy‐induced senescence
Treg: regulatory T cells
VEGF: vascular endothelial growth factor

## 1 Introduction

Cellular senescence is a phenotype of irreversible cell cycle arrest, and was originally discovered by Hayflick and Moorhead [1] in somatic cells that reached a finite lifespan following several passages. Cellular senescence was initially shown to function as a key mechanism of tumour suppression [2, 3]. More recently, however, the chronic and persistent influence of senescent cells on tissue homeostasis has attracted attention with the discovery of the senescence‐associated secretory phenotype (SASP) [4, 5, 6]. It has become apparent that SASP is often induced when the innate immune system activates NF‐κB‐associated and/or IFN‐associated signalling, both of which induce the expression of genes that encode a series of inflammatory factors [3, 7].

In this review, we provide an overview of cellular senescence and the SASP, particularly in relation to the potential clinical investigations of cancer therapies that utilise senescent cells. We also review the recently emerging role of the SASP in stromal as well as tumour cells in remodelling the tumour microenvironment, with emphasis on their context‐dependency that determines whether they promote or suppress cancer development.

## 2 Regulation of cellular senescence and the SASP

**Fig. 1:**
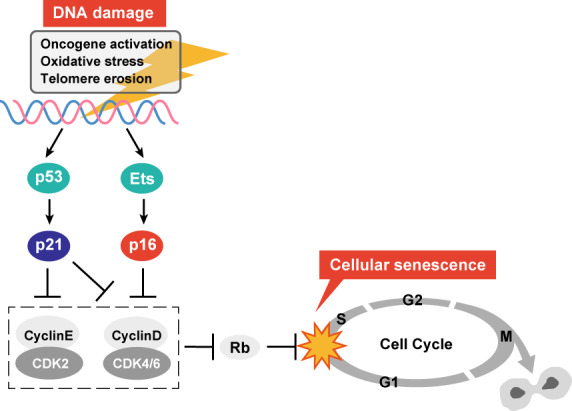
Central regulators of cellular senescence. p16 and p21 are upregulated upon DNA damage and are the most important CDKIs that contribute to senescence‐associated cell cycle arrest. p53 is the major transcription factor that mediates DNA damage‐induced p21 activation. These CDKIs suppress de‐repression of E2F transcription factors that is mediated by CDK‐dependent phosphorylation of E2F repressor Rb. Cyclin D1 is known to be upregulated in senescent cells but it is reported to be dysfunctional [160]. [Colour figure can be viewed at wileyonlinelibrary.com]

Cellular senescence is a state of permanent cell proliferation arrest [1] that can be induced by a variety of cellular stresses, including persistent DNA damage caused by, for example, oxidative stress and DNA replication errors (Box 1). When irreparable DNA damage persists, the expression of various p53‐target genes are induced, including cyclin‐dependent kinase inhibitor (*CDKI*), p21 (Cip/Kip family) and the INK4 family CDKI *p16*, which is upregulated by Ets family of transcription factors [8]. These CDKIs collaboratively and persistently arrest the cell cycle, which is the fundamental mechanism that induces cellular senescence (Fig. 1).

Box 1 **Types of cellular senescence**

Senescence can be classified at least into three subgroups; replicative senescence, oncogene‐induced senescence (OIS), and stress‐induced premature senescence (SIPS). Although these share many key regulatory mechanisms of senescence‐associated cell cycle arrest and all result in permanent cell cycle arrest, other senescence‐associated phenotype, such as the SASP, can be greatly different in these three different types of senescent cells. Cellular senescence that is induced after finite number of cell division in normal cells under normal condition is called replicative senescence. This is the most classical form of cellular senescence and is the one discovered by Hayflick and Moorhead [1]. At least in human fibroblasts *in vitro*, the major driver of replicative senescence is telomere shortening. Mouse fibroblasts and most epithelial cells *in vitro* undergo replicative senescence without critical telomere shortening, probably due to the activation of stress signals under nonoptimal culture condition [140, 141]. Especially, oxidative stress that is caused by ambient oxygen, which is higher than *in vivo*, can be the major driver of replicative senescence in mouse cells. High levels of oxidative stress or other genotoxic stress can trigger acute cellular senescence response that is called SIPS. Cellular senescence observed in normal ageing often harbours persistent DNA damage foci at telomere region [142]. It seems that this is because DNA damage formed at the telomere region is more difficult to repair and it is not because of telomere shortening. Surprisingly, it is still not clear what type of stress is responsible for the age‐related accumulation of senescent cells. OIS is a form of cellular senescence that is triggered by oncogene activation [143] and is a major anti‐tumour mechanism which also explains why cellular senescence machinery has evolved. Oncogene‐induced senescent cells are found in many precancerous lesions [144, 145, 146] and have significant effects on the tumour microenvironment and tumour development, as discussed in detail in this review. Cellular senescence induced by cancer therapy is called therapy‐induced senescence (TIS), which at least in some cases is identical to SIPS. Therapy‐induced senescent cells also have significant effects on the tumour microenvironment and tumour development. TIS/SIPS are a promising target for future cancer treatment.

**Fig. 2:**
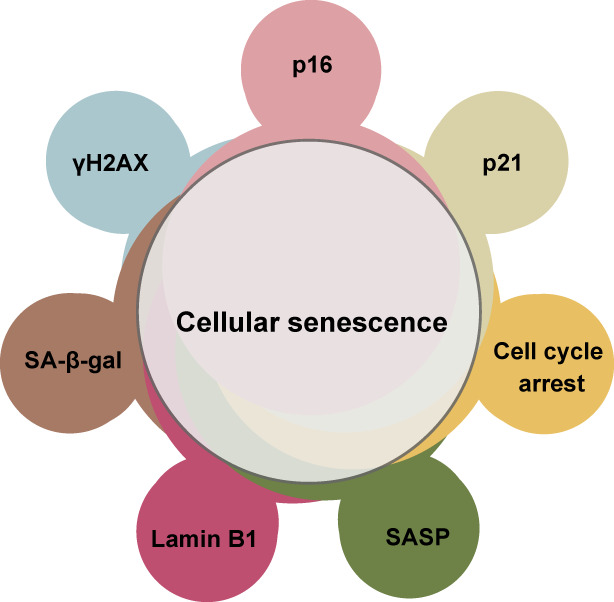
Markers of cellular senescence. Markers most commonly used to identify senescent cells include cell cycle arrest, p16, p21, Lamin B1 downregulation, γH2AX, SA‐β‐gal and the SASP. However, neither of these markers is truly specific to senescent cells. For example, quiescent cells also exhibit cell cycle arrest and, in some cases, increased SA‐β‐gal activity. It is important to combine several markers in a battery of tests in order to identify senescent cells. [Colour figure can be viewed at wileyonlinelibrary.com]

The *p53* and *p16* genes are also frequently inactivated in various human cancers [2], highlighting the importance of these senescence‐inducing pathways for tumour suppression. *p53* [9] and *INK4a (p16)* gene loci [10] are frequently mutated in human cancers. In addition, hypermethylation of the *p16* gene promoter that silences *p16* gene expression is often detected in human cancers [11], illustrating that these cancer cells have lost the cellular senescence‐inducing mechanism [12]. Senescent cells are not simply dormant, nonproliferating cells but are metabolically active and secrete a variety of proteins [13]. This phenotype is called the SASP, and is a very important trait of mature senescent cells, which are characterised by their increased secretion of numerous bioactive factors, including cytokines, chemokines, proteases and growth factors [14, 15]. More recently, bioactive lipid mediators, such as prostaglandins and exosomes, have been recognised as also being SASP‐associated factors [15, 16, 17]. The SASP can influence many types of cells that reside in the vicinity of senescent cells, including fibroblasts, immune cells, vascular endothelial cells and tumour cells, in a paracrine manner, activating signalling pathways in the tissue microenvironment [18]. SASP factors can also induce a variety of physiological outcomes, including beneficial effects such as tissue repair [19] and detrimental effects such as tumorigenesis [14], depending on the cell type, stimulus and biological context. Some of this diversity in cultured cells is summarised in the SASP atlas showing that distinct SASP factors are produced depending on cell types [20]. However, even more complex SASP outcomes might occur *in vivo* in the contexts of disease or ageing. Thus, targeting the SASP *in vivo* is an emerging but important aspect of senescence research, since it could lead to therapies for diseases including cancer [7]. The basic features of senescent cells are summarised in Box 2 and Fig. 2 as markers of senescent cells. However, it should be noted that none of these markers is solely specific to senescent cells. Therefore, it is important to combine several markers in order to precisely identify senescent cells.

Box 2 **Markers of cellular senescence**

While permanent cell cycle arrest is the most essential feature of cellular senescence, it is not easy to test whether the cell cycle arrest is truly irreversible. However, there are several other characteristics that are often, but not always, associated with cellular senescence and can be used in combination to identify senescent cells. Apart from cell cycle arrest, markers that most commonly used to identify senescent cells include p16, p21, DNA damage response marker γH2AX, downregulation of nuclear envelope protein lamin B [37], senescence‐associated β‐galactosidase activity (SA‐β‐gal) [147] and the SASP [5]. The induction of CDK inhibitors p16, p21 and DNA damage responses involving γH2AX play direct roles in permanent cell cycle arrest. Lamin B downregulation is thought to contribute to the SASP via leakage of DNA to cytoplasm and the SASP promotes the establishment of cellular senescence. SA‐β‐gal activity, which is expressed from lysosomal β‐d‐galactosidase, GLB1, seems unnecessary for the induction or maintenance of cellular senescence and its functional importance is not clear [148]. Note that, none of these markers is solely specific to senescent cells. For example, cell cycle arrest in quiescent cells can be reversible; p16 can be detected in several cancer cells, especially in HPV‐related cancer cells in which Rb is inactivated by HPV oncoprotein E7 independent of p16 [149]; p21 can be upregulated upon transient DNA damage; Lamin B1 can be downregulated independent of cellular senescence; γH2AX can be detected in G2/M phase cells; SA‐β‐gal activity can be increased in quiescent cells and several cancers; SASP‐like inflammatory response can occur independently of cellular senescence by innate immune responses in other types of cells. Therefore, it is important to combine several markers in a battery of tests in order to precisely identify senescent cells.

The induction of SASP factors, which include many inflammatory cytokines and chemokines, is regulated transcriptionally and post‐transcriptionally and requires the activation of NF‐κB, thereby eliciting an inflammatory cascade [6, 21]. IL‐1α plays a primary role in promoting NF‐κB signalling by upregulating many SASP factor genes [22]. The inflammasome, through which IL‐1β is activated, has also been shown to induce SASP factor genes [15, 23]. Other factors that can induce the expression of SASP factor genes include the transcription factors CCAAT/enhancer‐binding protein beta (C/EBPβ) [4, 24] and GATA binding protein 4 (GATA4) [25], particularly in oncogene‐induced senescent cells. For example, GATA4, whose levels increase during ageing or upon total body irradiation, is stabilised by the DNA damage response and has been shown to collaborate with NF‐κB to induce the expression of SASP factors at least *in vitro* [25]. C/EBPβ and c‐Myc also collaborate with NF‐κB to induce the expression of SASP factors [4]. In contrast, NOTCH1 can repress C/EBPβ activity and inhibit pro‐inflammatory SASP factor secretion in fibroblasts *in vitro* and *in vivo* [26]. Suppression of Janus kinase (JAK)/signal transducer and activator of transcription (STAT) pathway, that promotes cytokine expression, has been shown to alleviate the SASP *in vitro* and frailty *in vivo* in aged mice [27]. SASP factors are also regulated post‐transcriptionally, for example, by the RNA‐binding protein ZFP36L1, which stabilises SASP mRNA transcripts following the mTOR‐mediated activation of MK2 at least in cultured fibroblasts [28]. mTOR and p38MAPK [23] signalling have also been shown to play a crucial role in the regulation of SASP factor expression in cultured fibroblasts. Accordingly, the inhibition of the mTOR pathway by Rapamycin represses SASP factor expression [28].

**Fig. 3:**
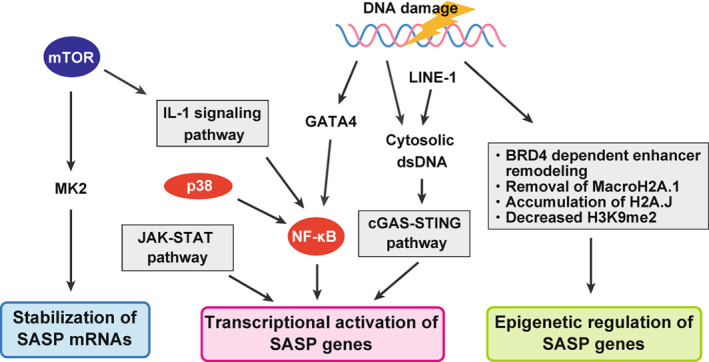
Central regulators of the SASP. SASP is regulated at epigenetic, transcriptional and post‐transcriptional levels in senescent cells. Major pathways involved in SASP induction are depicted in the figure. Many of these pathways are activated by DNA damage. [Colour figure can be viewed at wileyonlinelibrary.com]

The SASP is also epigenetically regulated [3, 29]. For example, our group has demonstrated that DNA damage response‐driven reduction of histone H3K9 di‐methylation in the promoter regions of key SASP factor‐encoding genes contributes to SASP factor de‐repression in cultured fibroblasts as well as in mouse skin papilloma *in vivo* [30]. Another example showed that pro‐inflammatory cytokines in a mouse model of gastric cancer downregulate EZH2, a histone H3K27 methyltransferase in senescent cancer‐associated fibroblasts (CAFs). This downregulation maintains SASP factor expression by demethylating H3K27me3 to enhance peritoneal tumour formation in gastric cancer through JAK/STAT3 signalling [31]. Moreover, the histone variants macro‐H2A.1 [32] and H2A.J, which accumulate in senescent cells, are also associated with SASP factor induction [33]. Furthermore, the chromatin reader BRD4 has been shown to remodel the enhancer landscape to activate the expression of key SASP factor genes in senescent cells [34]. Understanding how these factors and their upstream signals regulate the SASP might enable cancer treatment that targets the SASP, thereby remodelling the tumour microenvironment (Fig. 3).

## 3 SASP induction mediated by the innate immune response

**Fig. 4:**
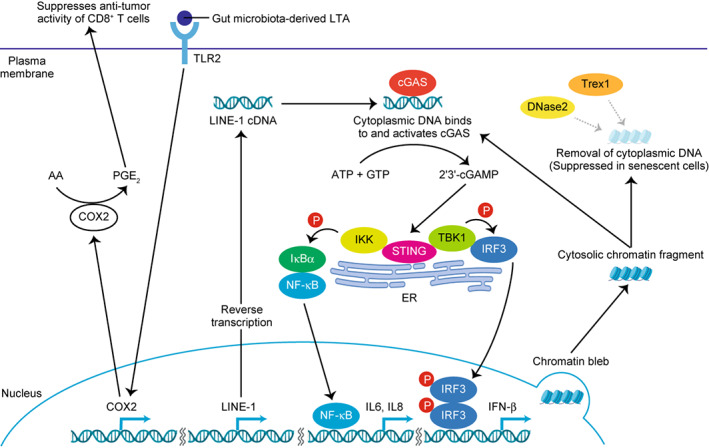
SASP‐related innate immune responses. Cytoplasmic DNA‐induced cGAS‐STING innate immune pathway plays a pivotal role in SASP induction. Mechanisms underlying the senescence‐associated increase in cytoplasmic DNA include impaired nuclear membrane integrity, cytoplasmic chromatin fragment formation, de‐repression of LINE‐1, and reduced expression of DNase2 and TREX1. Extrinsic inducers of innate immune responses also contribute to the SASP. For example, gut microbiota‐derived LTA stimulates TLR2 expressed on senescent hepatic stellate cells and thereby upregulates COX2 expression, which then produces immunosuppressive PGE_2_. [Colour figure can be viewed at wileyonlinelibrary.com]

Innate immunity has recently emerged as an important mechanism for inducing the expression of SASP factors. The cyclic GMP‐AMP synthase (cGAS)‐stimulator of interferon genes (STING) pathway plays an important role in the activation of type‐I interferon signalling in senescent cells, which is associated with subsequent SASP factor expression. cGAS‐STING pathway is triggered by DNA damage‐associated double‐strand DNA fragment, which has been identified as an important intrinsic mechanism for induction of a variety of SASP factors. However, *in vivo*, there should be many potential extrinsic triggers such as damage‐associated molecular patterns (DAMPs) and pathogen‐associated molecular patterns (PAMPs) including lipoteichoic acid (LTA) a cell wall component of gram‐positive bacteria, for SASP factor induction [15]. Moreover, as discussed below, innate immunity can be activated by ageing or obesity, which are both major risk factors for cancer. The prolonged inflammation in ageing and obesity is now understood as a concept of ‘inflammaging’, which is also triggered by ageing‐associated innate immune response. We discuss it in the last part of this section (Fig. 4).

### 3.1 Intrinsic mechanism: cGAS‐STING‐mediated regulation

Originally, cGAS was identified as a sensor of cytosolic DNA, which is recognised as being a potential indicator of an invading pathogen (since DNA typically exists only in the nucleus or mitochondria). The cytosolic DNA, from whatever origin, can be recognised as DAMP that triggers the innate immune response [35]. cGAS is a sensor that binds cytosolic DNA independently of DNA sequence to induce a conformational change in the catalytic unit of the cGAS enzyme that creates cyclic dinucleotides, such as cyclic GMP‐AMP (cGAMP) [36]. These cyclic dinucleotides act on STING, thereby inducing type‐I interferon signalling [36]. In senescent cells, DNA fragments are continuously released into the cytoplasm because of the fragility of the nuclear membrane and reduced lamin B1 expression [37]. At least part of these DNA fragments exists in the form of large cytoplasmic chromatin fragments (CCFs). CCFs are lamin A/C‐negative and strongly γH2AX‐positive, and are generated by a nucleus‐to‐cytoplasm blebbing of chromatin [38, 39, 40]. Normally, cytosolic DNA is degraded by DNases, such as DNase2 and TREX1, or by autophagy/lysosomal pathways [36, 41]. However, these DNases are downregulated in senescent cells, leading to the accumulation of cytosolic DNA fragments and the subsequent abnormal activation of the cGAS‐STING pathway [41]. Micronuclei also act as stimulators of the cGAS‐STING pathway. Micronuclei are small DNA aggregates often produced by chromosomal damage after genotoxic stress, and are observed in senescent cells [38, 42, 43].

In addition to these cytoplasmic factors, cDNA transcribed from the mRNA of LINE‐1 (LINE‐1 are retrotransposable elements that contain reverse transcriptase genes) also accumulates in the cytoplasm of senescent cells [44]. LINE‐1 is transcriptionally de‐repressed in senescent cells and activates a type‐I interferon (IFN‐I) response, contributing to the persistence of the SASP through the cGAS‐STING pathway [44]. These events are observed in many tissues of aged wild‐type mice [45]. However, when aged mice are treated with an inhibitor of nucleoside reverse transcriptase, IFN‐I activation and age‐associated inflammation (inflammaging) are both downregulated in various tissues [45]. Increased LINE‐1 cDNA has also been observed in SIRT6‐deficient mice, which show accelerated ageing [45]. Together, these studies suggest that the abnormal accumulation of cytosolic DNA stimulates the cGAS‐STING pathway, thus contributing to the induction of the SASP. Other innate immune receptors, such as TLR1 [46] and TLR2 [47] are also reportedly associated with cellular senescence and the SASP via their ligand‐mediated signalling and can be important SASP regulators in tumour microenvironment as discussed in detail below.

### 3.2 Extrinsic SASP induction via gut‐derived MAMPs and PAMPs

Microbial‐associated molecular patterns (MAMPs) and PAMPs are innate immune‐activating molecules that derive from microbiota and pathogen, respectively, and are linked to senescence and SASP because senescent cells are highly sensitive to innate immune responses. Both intrinsic (e.g. host‐derived cytoplasmic DNA) and extrinsic ligands (e.g. gut microbiota‐derived lipoteichoic acid) that stimulate innate immunity can promote the SASP. Our group has previously shown that deoxycholic acid (DCA), an obesity‐associated gut microbial metabolite, induces cellular senescence and the tumour‐promoting SASP in mouse hepatic stellate cells (HSCs) by persistent DNA damage due to reactive oxygen species (ROS) production in the obesity‐associated liver tumour microenvironment [48]. In high‐fat diet (HFD)‐fed mice, gram‐positive gut microbiota abundance is greatly increased, and lipoteichoic acid (LTA), a cell wall component of gram‐positive bacteria, accumulates in their livers, triggering the SASP, especially in the liver tumour microenvironment. HFD‐fed mice lacking TLR2, a receptor that recognises LTA, develop significantly fewer liver tumours than HFD‐fed wild‐type mice. LTA‐TLR2 signal activates COX2 in DCA‐induced senescent HSCs and thereby enhances prostaglandin E_2_ production, which then suppresses the anti‐tumour function of CD8^+^ T cells. Thus, LTA, derived from gram‐positive gut microbiota in HFD‐fed mice, acts as an extrinsic factor for SASP induction. PGE_2_, as a SASP factor generated by senescent HSCs, is crucial for suppressing anti‐tumour immunity mediated by CD8 T lymphocytes [15]. COX‐2 upregulation and the overproduction of PGE_2_ have also been observed in human nonalcoholic steatohepatitis‐associated liver cancer [15]. Moreover, TLR2 [47, 49] and TLR4 [50] are also reportedly activated in senescent cells, indicating their potential involvement in the extrinsic control of SASP induction [50].

### 3.3 Inflammaging

Chronic inflammation, caused by ageing and obesity, can promote cancer progression in many different ways [51], for example, by altering cell proliferation, cell survival, cell renewal, epithelial‐mesenchymal transition (EMT), angiogenesis, migration and immunosuppression, similarly to the SASP. Low‐grade, chronic and sterile systemic inflammation, called inflammaging, is a feature of ageing and obesity [52] and is associated with the SASP in the senescent cells of aged organisms. The age‐related activation of inflammatory genes has been observed in many tissues in both mice and humans [53, 54, 55, 56]. For example, NF‐κB, a pro‐inflammatory transcription factor that plays a key role in the SASP, reportedly drives age‐related gene expression changes in numerous mouse and human tissues [57], implicating the role of senescent cells in age‐related, inflammatory gene activation. Some inflammatory factors can also be inhibited with senolytic drugs that specifically kill senescent cells. For example, when the senolytic drugs, dasatinib and quercetin, were given to mice in combination, they suppressed the age‐associated upregulation of the cytokine IL‐6 in adipose tissue [58]. It should be noted that age‐related increases in DAMPs, MAMPs and PAMPs can also trigger the systemic activation of NF‐κB and inflammation [59]. Ageing also diminishes apoptotic cell clearance [60], and immunogenic mtDNA increases in the blood with age, both of which produce DAMPs to trigger innate immunity [61]. Increased levels of lipopolysaccharide (LPS)‐binding protein, a surrogate marker for LPS and other bacterial products, are also found in the blood of the elderly, and indicate gut barrier dysfunction [62]. All these factors can activate NF‐κB and inflammation. Age‐related immune dysfunction, namely immunosenescence, is also likely to play a role in inflammaging as discussed later in this review [63]. In conclusion, inflammatory status of the microenvironment, which is highly context‐dependent, is a crucial upstream regulator of the SASP.

## 4 Context‐dependent effects in the tumour microenvironment

**Fig. 5:**
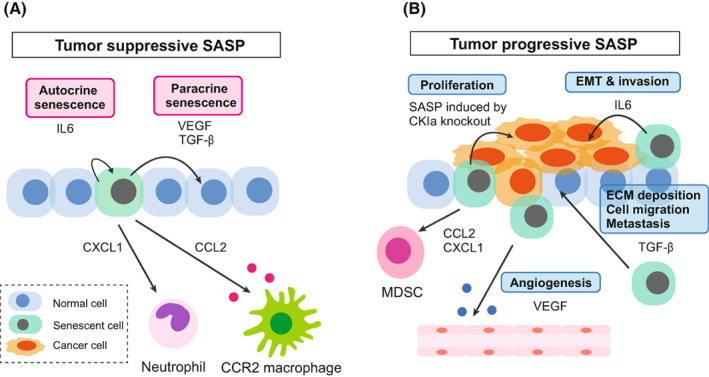
The context‐dependent roles of SASP in tumour microenvironment. (A) Tumour‐suppressive SASP. In normal or precancerous tissue, senescent cells have a tumour‐suppressive role by re‐enforcing senescence through the SASP, which has both autocrine and paracrine effects. SASP factors can recruit immune cells to clear themselves (senescence surveillance). (B) Tumour progressive SASP. In advanced cancer tissue, SASP factors produced by senescent cells can promote cancer progression by enhancing angiogenesis, cancer cell proliferation, EMT and the metastasis of cancer cells. SASP factors also suppress anti‐tumour immunity. [Colour figure can be viewed at wileyonlinelibrary.com]

The impact of SASP on the tissue microenvironment *in vivo* varies depending on the types of cells undergoing senescence (e.g. any of the stromal or epithelial cells, and normal or cancer cells can be senescent), and the cause of senescence (see Box 1), or the trigger of the innate immune pathway for SASP (e.g. cGAS‐STING, TLRs, etc.). The SASP not only reinforces cellular senescence in an autocrine manner [4] but also mediates its paracrine effects. SASP factors can remodel tissues in a paracrine manner, for example, by altering the ability of proliferation and migration of adjacent cells, such as stromal cells, immune cells and cancer cells [3, 14, 64]. SASP factors can also promote angiogenesis and enhance the immunosuppressive microenvironment [65, 66]. Overall, the complexity of the context‐dependent effects of SASP on the tissue microenvironment highlights the importance of identifying cells that produce specific SASP factors for therapeutic targeting [14, 29] (Table 1, Fig. 5A,B).

**Table 1:** The role of senescent cells in tumor microenvironment.

	Type of cancer	Senescent cell	Senescence inducer	Major role(s) of the SASP	SASP factor(s)
Anti‐tumorigenic SASP	Hepatocyte	Hepatoocyte	OIS (N‐Ras)	Immune‐mediated senescent cell clearance	IL‐1α [71]
	Lymphocyte	Lymphocyte	TIS (cyclophosphamide)	Reinforce cellular senescence	ND [21]
	Melanocyte	Melanocyte	TIS (AURKA or CDK4/6 inhibitor)	Lymphocyte recruitment	CCL5 [150]
	Melanocyte	Melanocyte	TIS (Aurora inhibitor)	Reinforce cellular senescence	ND [151]
	Osteoblast	Osteoblast	TIS (Irradiation)	NKT cell recruitment	IL‐6 [152]
	Pancreatic ductal cell	Pancreatic ductal cell	TIS (MEK and CDK4/6 inhibitors)	Increased vascularization and improved drug delivery efficacy	VEGF [15]
				Endothelial cell activation followed by accumulation of CD8^+^ T cells	CCL5, CXCL1, IL‐6 [15]
	Hepatocyte	Hepatocyte	OIS (N‐Ras)	Myeloid cell recruitment followed by macrophage differentiation under precancerous environment	CCL2 [70]
Pro‐tumorigenic SASP	Hepatocyte	Hepatocyte	OIS (N‐Ras)	Myeloid cell recruitment followed by MDSC differentiation under cancerous environment	CCL2 [70]
	Hepatocyte	Hepatic stellate cell	HFD‐induced senescence	ND	IL‐1β [48]
				Impairment of antitumor functions of CD8^+^ T cells	PGE_2_ [15]
	Lymphocyte	Lymphocyte	TIS (doxorubicin)	Stemness induction	ND [153]
	Mammary epithelial cell	Mammary epithelial cell	TIS (doxorubicin)	Mitogenic support	Eotaxin, CXCL5, Rantes [154]
	Mammary epithelial cell	Fibroblast	DNA damage (bleomycin)	Promotion of cancer invasion	MMPs [155]
	Mammary epithelial cell	Mammary epithelial cell	OIS (HER2)	Metastasis support	ND [156]
	Melanocyte	Fibroblast	TIS (CDK4/6 inhibitor)	Myeloid cell recruitment	ND [110]
	Mesothelial cell	Mesothelial cell	TIS (pemetrexed)	EMT induction and chemoresistance	ND [157]
	Prostate epithelial cell	Prostate epithelial cell	TIS (PTEN loss)	Myeloid cell recruitment followed by reinforcement of senescence	CXCL1, CXCL2 [158]
	Prostate epithelial cell	Prostate epithelial cell	TIS (PTEN loss)	MDSC recruitment	ND [121]
	Thyroid follicular cell	Thyroid follicular cell	OIS (BRAF)	Anoikis resistance	CXCL12 [159]

It is important to note that it is not only the constituents of the SASP are context‐dependent but that even a single SASP factor can be pro‐ or anti‐tumorigenic depending on the biological context. For example, the most renowned SASP factor, IL6, helps in establishing cellular senescence through an autocrine effect [6], and can also promote EMT and invasion of cancer cells *in vitro* [5]. SASP induced by CKIα knockout in intestinal epithelial cells also contribute to reinforcing cellular senescence in normal cells; however, it promotes cell proliferation of p53‐mutated precancerous cells [67]. VEGF and TGF‐β secreted by senescent cells can induce cellular senescence of the surrounding normal cells at least *in vitro* [18]. In the tumour microenvironment, however, VEGF secreted by senescent cells may promote angiogenesis [68] and TGF‐β secreted by senescent cells may promote extracellular matrix (ECM) deposition, cell migration, and metastasis [69]. CCL2 secreted by senescent hepatocytes recruits CCR2^+^ myeloid cells, which then become mature and eliminate senescent cells and thereby suppress cancer development. However, this maturation is blocked under the presence of abundant HCC cells and in this case CCR2^+^ myeloid cells promote cancer progression by suppressing NK cells [70].

### 4.1 Senescence surveillance in precancerous cells

**Fig. 6:**
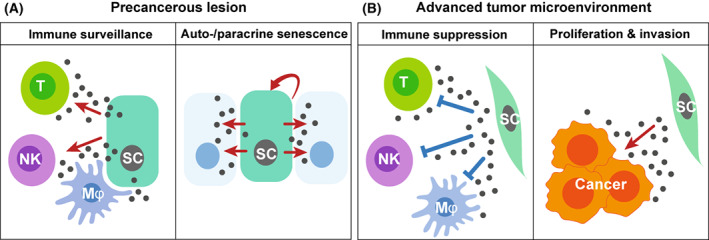
The effects of the SASP depends on tumour stage. (A) In precancerous tissues, the effects of the SASP are predominantly tumour‐suppressive, with the major tumour‐suppressive effects including autocrine and paracrine senescence and induction of immunosurveillance. (B) In advanced cancerous tissues, the SASP factors from stromal cells such as CAFs can promote tumour growth. [Colour figure can be viewed at wileyonlinelibrary.com]

Cellular senescence is an important tumour suppression mechanism (see Fig. 5A), and when the senescence‐inducing machinery in epithelial cells is disrupted, it can lead to the onset of cancer. Another important tumour prevention mechanism mediated by senescence is the clearance of premalignant cells when they undergo cellular senescence [71, 72]. This clearance system for senescent cells is called ‘senescence surveillance’ (Fig. 6A). Premalignant, senescent hepatocytes, which are transduced with oncogenic *N‐ras in vivo* have been shown to be cleared by an antigen‐specific immune response, which limits the development of liver cancer [70, 71]. This suggests that precancerous cells with senescent cell features tend to be eradicated by immune cells recruited by SASP factors. Indeed, a recent study has shown that senescent cells can be cleared by CAR‐T cells, which recognise a senescent cell‐specific cell‐surface marker [73].

## 5 Senescent cancer‐associated fibroblasts in the tumour microenvironment

Tumour tissues consist of cancer cells and many other cell types, such as immune cells, stromal cells and vascular epithelial cells. Among these, CAFs have recently emerged as a cell type that promotes cancer cell expansion in the tumour microenvironment. CAFs are often senescent and are associated with the inflammatory SASP (Fig. 6B) [74, 75]. Inflammatory CAFs in the tumour microenvironment have recently been designated iCAFs [75], and are similar to senescent CAFs exerting SASP [76, 77, 78]; myofibroblastic CAFs have been designated myCAFs [76, 78]. MyCAF populations display ligand‐receptor interactions that are distinct from those of iCAFs. ICAFs appear to be more tumour‐promoting than myCAFs by producing chemokines and cytokines [79] showing a higher malignancy in pancreatic tumorigenesis [80]. On the other hand, myCAFs may produce ECM extensively to impede drug delivery despite being less cancer‐promoting [81]. iCAFs and myCAFs seem to exclusively act on the surrounding cells.

Senescent CAFs in the tumour microenvironment often play a role in tumour progression. For example, in HFD‐fed mice, the entero‐hepatic circulation of DCA elicits DNA‐damage‐associated cellular senescence and the SASP in HSCs [15, 48]. In HFD‐fed mice that lack IL‐1β, an upstream regulator of the cytokine cascade, HSCs in the liver developed a senescence phenotype but show reduced expression of SASP factors [48]. These mice also showed a decline in liver tumour formation, suggesting that the IL‐1β‐mediated pathway in HSCs plays a role in obesity‐associated liver tumour progression. These results suggest that senescent HSCs function as senescent CAFs and play a key role in obesity‐associated liver cancer development through the secretion of SASP factors.

Radiotherapy and chemotherapy can both cause therapy‐induced senescence (TIS) (see Box 1) due to the DNA damage induction in tissue‐resident fibroblasts, which then become senescent CAFs via the DNA damage response [75, 82]. These senescent CAFs are often resistant to chemoradiotherapy and have a SASP phenotype that induces the production of pro‐tumorigenic factors, such as IL‐6 and IL‐8, which are associated with immunosuppression and with stroma‐mediated therapeutic resistance [83, 84, 85, 86, 87, 88]. TIS in stromal cells has been reported to cause undesirable roles such as breast cancer metastasis and therapy resistance in mouse models [83, 84]. In addition, senescent CAFs have been observed in the microenvironment of colon [89] and pancreatic tumours in mice, with p38 MAPK signalling inducing the pro‐tumorigenic SASP [90]. The upregulation of IL‐8, a well‐known SASP factor, in senescent CAFs permits pancreatic ductal adenocarcinoma (PDAC) cells to invade or metastasise [91, 92]. The presence of senescent CAFs have also been associated with reduced survival in patients with early stage of pancreatic tumours, showing their effect on cancer prognosis [92], and senescent CAFs also promote EMT and pancreatic cancer progression in mice [93]. As previously mentioned, inflammation (e.g. persistent cytokine secretion) itself can induce cellular senescence and the SASP via paracrine manner. Intriguingly, a recent study suggested that such sequence and persistence of SASP has also been associated with long Covid‐19 syndrome [94].

## 6 CDK4/6 inhibitors and SASP

**Fig. 7:**
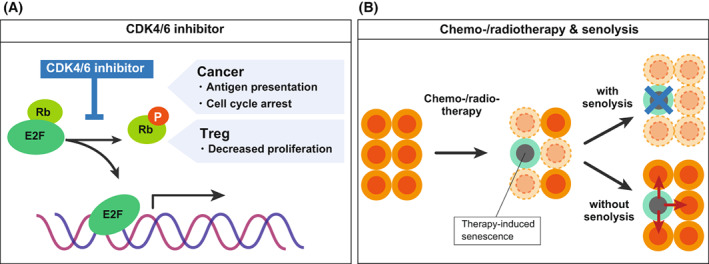
SASP in cancer therapy. (A) Schematic illustration of the effect of CDK4/6 inhibitor. (B) Schematic illustration of how the combination of chemotherapy and senolysis contribute to cancer therapy. [Colour figure can be viewed at wileyonlinelibrary.com]

The activation of cyclin D and CDK4/6, which play pivotal roles in the transition from G1 to S phase, is a feature of many cancer cell types, particularly breast cancer cells [95, 96, 97], and so both have been targeted therapeutically. For example, CDK4/6 inhibitors, such as palbociclib, abemaciclib, and ribociclib, are used clinically to treat oestrogen receptor‐positive (ER^+^) and human epidermal growth factor receptor 2‐negative (HER2^−^) breast cancer [98]. Since CDK4/6 inhibitors mimic the function of p16 [99], the induction of cellular senescence is a likely outcome of this treatment (Fig. 7A). Indeed, these CDK4/6 inhibitors have been shown to induce cellular senescence in many cancer types such as breast cancer (*in vitro* and *in vivo*), Ewing sarcoma (*in vitro*), and neuroblastoma (*in vitro*) [100, 101, 102, 103, 104, 105, 106], although in these studies senescence induction has mainly been judged by SA‐β‐galactosidase positivity, which can be an indicator of cellular senescence but is not an absolute marker solely because it is also known to be positive in some quiescent cells [107].

CDK4/6 inhibitors not only induce tumour cell cycle arrest but also promote anti‐tumour immunity, as they upregulate the expression of endogenous retroviral elements in tumour cells, producing increased intracellular levels of double‐stranded RNA, thereby stimulating the production of type III interferons, which in turn enhance tumour antigen presentation [100]. In addition, CDK4/6 inhibitors strongly suppress the proliferation of Treg cells. Eventually, these phenotypic changes promote the cytotoxic T‐cell‐mediated clearance of tumour cells, enhances the efficacy of cancer therapy by adding the treatment using immune checkpoint blockade. Other reports also suggest that premalignant senescent cells induced by CDK4/6 inhibition do not acquire pro‐tumorigenic and detrimental properties in patients with breast cancer patients, suggesting that these effects could be beneficial for senescence surveillance [100]. Senescence‐inducing CDK4/6 inhibitors when combined with other types of therapies have also been shown to be effective in a mouse model of PDAC. In this mouse model, CDK4/6 inhibitors in this mouse model induced the SASP in PDAC cells, increasing vascularity and the immune response in the PDAC tumour microenvironment. This initial microenvironmental remodelling improved the efficacy of VEGF inhibitors and immune checkpoint blockade [64]. SASP‐mediated improvement of immune checkpoint blockade efficacy was also observed in a topoisomerase 1 inhibitor‐ and cisplatin‐treated senescent ovarian cancer model [108, 109]. These findings suggest that TIS can promote immunotherapy. It should also be noted, however, that senescence of fibroblasts caused by prolonged exposure to CDK4/6 inhibitor can accompany the SASP that increases and decreases the population of Gr‐1‐positive immune cells and CD3‐positive cells in the tumour microenvironment, respectively, and promotes melanoma growth in mice due to the suppression of anti‐tumour immunity [110]. Therefore, although CDK4/6 inhibitors are promising agents for the future senescence‐based cancer therapy, further investigation is warranted to establish their effective usage.

## 7 Senolysis and senomorphics for cancer therapies

Overall, senescent cells exert detrimental effects on the tumour microenvironment, particularly SASP‐induced senescent cells in advanced cancers (Fig. 7B) [14]. Given this, senolytic drugs are currently being developed to target senescent cells to eliminate their deleterious effects [111, 112]. Senescent cells cease proliferating, exhibit DNA damage response signals, but remain alive by avoiding apoptosis [112]. This is a key trait of cellular senescence, which can be targeted for therapeutic purposes. Dasatinib and quercetin were the first‐identified combination of senolytic drugs that could reactivate suppressed apoptotic signals in senescent cells [113]. Inhibitors of B‐cell lymphoma‐2 (BCL‐2)/BCL‐XL have also been developed as senolytic drugs [114]. Another recently identified senolytic drug is the BET family protein degrader (BETd), which induces senolysis via two independent pathways: through the inhibition of nonhomologous end‐joining (NHEJ) and the upregulation of autophagic gene expression [115]. In senescent cells, only NHEJ is available as a double‐stranded DNA break repair system due to the cell cycle arrest that occurs in cellular senescence. Autophagy is also suppressed in senescent cells and is derepressed by BETd to induce cell death by autophagic gene upregulation [115]. BETd treatment has been shown to effectively suppress liver cancer in mice [115]. Another recently reported and effective senolytic drug is an inhibitor of glutaminase 1 (GLS1), which targets the altered metabolic fragility of senescent cells [116]. Immune‐based strategies for eliminating senescent cells have also been reported, such as targeting the senescent cell‐specific surface marker, uPA receptor, by generating chimeric antigen receptor re‐directed (CAR) T cells [73], or by creating a senolytic vaccine that targets both CD153 on senescent T cells [117] or glycoprotein nonmetastatic melanoma protein B (GPNMB) in senescent endothelial cells [118].

A two‐step approach has been proposed for using senolysis to target cancer cells: first, cellular senescence is induced in cancer cells, and then senescent cancer cells are eliminated via senolysis. The senolytic drug, ABT263, has been used to successfully treat chemotherapy‐induced senescence in cancer cells in a breast and lung cancer xenograft model. Its administration after treatment, together with either etoposide or doxorubicin (DNA‐damaging chemotherapeutic agents), resulted in prolonged tumour suppression in tumour‐bearing animals [119]. Similar success was achieved by using a senolytic drug, ABT263, in a xenograft model to eliminate senescent malignant meningioma cells induced by gemcitabine treatment followed by ionising radiation [120]. Senolytic therapy following conventional cancer therapy could thus improve therapeutic outcomes and effectively delay disease recurrence.

Since the SASP seems to be mediating many deleterious effects of senescent cells, suppression or modulation of the SASP instead of killing senescent cells could be another promising approach for targeting senescence‐associated diseases. Compared to senolytic drugs, drugs targeting the SASP, namely ‘senomorphics’, may only have transient effects but will also have lower cytotoxicity. Moreover, senomorphic drug can even convert ‘bad senescent cells’ into ‘good senescent cells’. For example, JAK2 inhibitor can reprogram immunosuppressive pro‐tumorigenic SASP into inflammatory anti‐tumorigenic SASP [121]. Drugs that have been shown to extend animal lifespan, such as rapamycin [122] and metformin [123], can also suppress SASP. These drugs are also known as anti‐cancer drugs, suggesting that senomorphic effects might contribute to anti‐cancer effects.

## 8 Immunosenescence is associated with impaired anti‐tumour immunity

Cellular senescence occurs in human T‐cells, causing the immune system to become dysregulated during normal ageing [124]. This is known as immunosenescence and it plays an important role in tumour development [125, 126]. In humans, the thymic generation of new naive T cells dramatically decreases by middle age [127, 128] and together with the age‐associated clonal expansion of T cells, diminishes the T‐cell receptor repertoire [128]. Notably, the incidence and prevalence of cancer also increase with age, possibly due to an increase in senescent T‐cell population in aged people [129, 130].

Senescent CD8^+^ T‐cell population increase in aged people, in younger individuals with chronic viral infections, and in patients with certain types of cancers [124, 131]. Moreover, Treg cells (natural Tregs or nTregs, and tumour‐derived Tregs), as well as tumour cells, suppress the function of naïve and effector T cells by inducing T‐cell senescence [126, 132, 133, 134]. These senescent T cells exhibit an altered phenotype that exerts suppressive activity on anti‐tumour immunity in the tumour microenvironment. A recent study has found that T‐cell senescence, induced by Tregs and tumour cells, is mediated by the dysregulation of lipid metabolism and can be reversed pharmacologically. This study also showed that normalising lipid metabolism and reversing senescence reduced tumour progression and extended survival in mouse models of melanoma and breast cancer [135].

Senescent T cells are metabolically active and have a unique SASP, which influences both immune cells and tumour cells in the tumour microenvironment. Recent studies indicate that senescent T cells induced by Treg and tumour cells can secrete proinflammatory cytokines such as IL‐6, IL‐8 and TNFα [133, 134, 136]. These cytokines can induce the premature senescence of the surrounding cells via paracrine mechanisms [4, 6, 137, 138], which can induce more senescent T cells in the tumour microenvironment that provide suppressive anti‐tumour immunity. Importantly, high levels of senescent CD8^+^ T cells predict poor prognosis in several types of cancer, such as lung cancer, gastric cancer, renal cell carcinoma, glioblastoma, non‐Hodgkin lymphoma, chronic lymphocytic leukaemia and acute myeloid leukaemia [139].

## 9 Conclusion and perspectives

The molecular mechanisms of cellular senescence and SASP have long been investigated using cultured cells. However, increasing evidence suggests that the phenotypes of senescent cells, including the SASP, might significantly differ *in vitro* and *in vivo*. Clarifying the role of cellular senescence *in vivo*, particularly in the tumour microenvironment, is crucial for a better understanding of cancer development and cancer therapy. As mentioned, the role of the SASP is highly context‐dependent; therefore, elucidating the precise mechanism of cellular senescence and SASP *in vivo* and the development of appropriate senolysis/senomorphic drugs in each case could lead to the establishment of means for cancer prevention and cancer therapy. In this regard, it is particularly important not to forget that the SASP is not necessarily harmful in the context of cancer treatment. In such situation, it could be better to alter the SASP factors rather than just suppress it, to promote immune surveillance, for example. Future studies should enable an appropriate usage of senolytics and senomorphics depending on cancer types. Regarding senolytics, it would be important to investigate its long‐term impact on tissue turnover. It might possibly occur that the removal of senescent cells increases the burden on the rest of the cells, which could lead to tissue dysfunction eventually. Further investigation of the effects of senolytics and senomorphics together with a better understanding of context‐dependent effects of the SASP *in vivo* would lead to the development of effective therapy for age‐related diseases including cancer.

## Conflict of interest

The authors declare no conflict of interest.

## Author contributions

NO contributed to conceptualisation; MT, YY and NO contributed to writing – original draft; NO and MT contributed to writing – review and editing and funding acquisition.

## Data Availability

Original data is not provided in this review paper.
